# Impact of Monaco sequencing parameters on monitor units, plan quality, and optimization time for Elekta Unity liver SBRT plans

**DOI:** 10.1002/acm2.70547

**Published:** 2026-03-18

**Authors:** Bryan C. Bates, Guanghua Yan, Amanda Schwarz

**Affiliations:** ^1^ Department of Radiation Oncology University of Florida Gainesville Florida USA

**Keywords:** Monaco, SBRT, sequencing parameters, treatment planning, unity

## Abstract

**Background:**

Magnetic resonance‐guided radiation therapy (MRgRT) using the Elekta Unity MR‐linac offers significant advantages for liver stereotactic body radiation therapy (SBRT). However, the Unity is limited by a low dose rate and the lack of volumetric‐modulated arc therapy (VMAT), resulting in prolonged treatment times. Reducing monitor units per fraction (MU/Fx) by optimizing Monaco sequencing parameters may improve treatment efficiency.

**Purpose:**

To evaluate the impact of Monaco sequencing parameters on MU/Fx, plan quality, and optimization time for Unity liver SBRT.

**Methods:**

Ten liver SBRT patients previously treated on the Unity were replanned. For each patient, 33 plans were generated by varying one of five sequencing parameters: maximum number of segments per plan, minimum MU per segment, minimum segment width (MSW), minimum segment area (MSA), and fluence smoothing (FS). The MU/Fx, optimization time, and estimated delivery time were recorded for each plan. Dosimetric and hotspot constraint compliance and the RTOG 0915 conformality index (CI) and gradient index (RI) were used to assess plan quality.

**Results:**

Reducing the maximum number of segments to no fewer than 30 and increasing the MSA produced the largest reductions in MU/Fx (3.4%–50.8% and 3.7%–43.2%, respectively) for all patients’ clinically acceptable plans. Using a high FS yielded modest MU/Fx reductions (3.0%‐17.6%) in eight patients. Minimum MU per segment and MSW showed negligible effects on MU/Fx among clinically acceptable plans. MSWs of 1.5 cm or greater resulted in degraded plan quality or clinically unacceptable plans. For the five patients with the largest planning target volumes (PTVs), optimization time decreased with fewer segments (1.1%–416%) and with increased MSA (22.5%–48.1%). Across all patients, optimization time decreased with increasing minimum MU per segment (3.0%–47.2%). Estimated delivery time strongly correlated with MU/Fx (*R^2^* = 0.8278).

**Conclusions:**

Adjusting Monaco sequencing parameters—particularly lowering the maximum number of segments, increasing the MSA, or using a high FS—can reduce MU/Fx and treatment time while maintaining acceptable plan quality on a patient‐specific basis.

## 1 INTRODUCTION

Magnetic resonance‐guided radiation therapy (MRgRT) has emerged as a safe and effective approach for treating liver tumors, particularly when delivered with stereotactic body radiation therapy (SBRT).^1^, ^2^, ^3^, ^4^, ^5^ MRgRT is uniquely suited for liver SBRT due to the several advantages it offers over conventional radiotherapy including superior soft tissue contrast from radiation‐free magnetic resonance (MR) imaging, the ability to perform daily adaptive planning, and real‐time imaging for respiratory gating.^1^, ^2^, ^3^, ^4^, ^5^

The Elekta Unity MR‐linac equipped with Comprehensive Motion Management (CMM) software benefits from all the advantages of MRgRT, but its design introduces unique challenges to treatment efficiency. To accommodate the MR components positioned between its radiation‐producing components and bore axis, the Unity employs an extended source‐to‐axis distance of 143.5 cm.^6^, ^7^, ^8^ Consequently, despite using a 7 MV flattening filter‐free (FFF) beam, the Unity is limited to a nominal dose rate of 425 monitor units (MU) per minute, with a maximum reported dose rate of 500 MU/min.^3^, ^6^, ^7^, ^8^, ^9^ This is substantially lower than that of systems such as the Varian TrueBeam STx which achieves up to 1400 MU/min with a 6 MV FFF beam.^10^ Furthermore, although the Unity hardware can support volumetric‐modulated arc therapy (VMAT), clinical delivery is currently limited to step‐and‐shoot intensity‐modulated radiation therapy (IMRT).^9^ These IMRT deliveries are even further restricted since the Monaco treatment planning system forbids beam angles that pass through the cryostat crossover pipe before reaching the patient.^11^, ^12^ Additionally, treatment plans are often designed to avoid using beam angles that would similarly traverse the high‐Z materials of the Unity's couch.^11^, ^12^ This is all despite preliminary analyses by Kontaxis et al. suggesting that VMAT on the Unity could reduce treatment times by up to 50% compared to IMRT while still avoiding irradiation of the cryostat crossover pipe.^9^

The limited dose rate and the lack of VMAT functionality prolong treatment delivery on the Unity.^10^, ^13^ When CMM gating is additionally applied, treatment delivery times are further increased due to duty cycles that can be as low as 41% for upper abdominal tumors in patients with irregular breathing patterns or image registration issues.^14^ Long treatment delivery times reduce patient comfort, limit patient throughput, and may compromise treatment efficacy.

A potential means of reducing treatment delivery time is to minimize the number of MU delivered per fraction (MU/Fx). Since the Unity's multi‐leaf collimator (MLC) achieves an effective maximum leaf speed of 6.5 cm/s–comparable to the Elekta Agility MLC–through the combination of individual leaves moving at 3.5 cm/s and the entire leaf bank moving at 3.0 cm/s, the MU/Fx provides the largest contribution to the treatment delivery time.^6^, ^15^, ^16^ Thus, lowering the MU/Fx may prove an effective method of decreasing treatment delivery time. To this end, prior work has shown that adjusting IMRT sequencing parameters can reduce MU/Fx by limiting plan complexity.^17^, ^18^ In the Monaco treatment planning system, sequencing parameters influence complexity as follows: (1) the maximum number of segments directly restricts the number of segments per plan, (2) the minimum monitor units per segment (MU/segment) sets the lowest deliverable MU for each segment, (3) the minimum segment width (MSW) defines the smallest permissible distance between opposing leaves in each segment, (4) the minimum segment area (MSA) establishes the smallest allowable field size per segment, and (5) fluence smoothing (FS) modifies the initial fluence map to reduce fluctuations and improve uniformity.

Although the Unity has been in clinical use for several years, to our knowledge, no published studies have evaluated how Monaco sequencing parameters affect MU/Fx for Unity IMRT plans. Some studies, however, have investigated these effects on conventional linacs. Hong et al. reported an 18.8% reduction in MU/Fx for esophageal IMRT when MSW was increased from 0.5 to 1.5 cm.^19^ Jimenez‐Puertas et al. found that increasing FS from off to high reduced MU/Fx by 5.1% for prostate IMRT, while increasing MSW from 0.5 to 1.0 cm reduced MU/Fx by 29% for gynecological IMRT.^20^ Finally, Wang et al. observed a 27.45% reduction in MU/Fx for left‐sided breast IMRT when MSW was increased from 0.5 to 1.5 cm.^21^ To date, no studies have examined the effects of maximum number of segments, minimum MU/segment, or MSA on the MU/Fx for IMRT plans in Monaco, nor has any study evaluated the impact of any Monaco sequencing parameter on the MU/Fx for liver SBRT.

Importantly, treatment delivery time is not the only factor that influences the overall treatment time on the Unity. Online plan optimization time is also critical.^22^, ^23^ Since plan optimization depends on the values set for each sequencing parameter, modifications to these parameters will affect the time required to complete the optimization.^24^ Similarly, plan quality will be affected by any changes in sequencing parameters that affect plan complexity.^17^, ^18^ As a result, the impact on plan optimization time and plan quality must be considered when varying the Monaco sequencing parameters for Unity plans.

In this work, we systematically evaluate how variations in individual Monaco sequencing parameters impact MU/Fx, plan quality, and plan optimization time for liver SBRT on the Elekta Unity with the goal of identifying parameter modifications that meaningfully reduce overall treatment time without compromising plan quality.

## 2 MATERIALS AND METHODS

### 2.1 Patient selection

Ten liver SBRT patients previously treated on an Elekta Unity MR‐Linac (Elekta AB, Stockholm, Sweden) before the integration of CMM software were selected for this study. Four patients were treated with 48 Gy in four fractions, while the remaining six were treated with 40 Gy in five fractions. Liver cancer was the primary cancer for two patients and a secondary cancer for the remaining eight. Although tumor locations varied among patients, all patients were treated with their arms down by their sides. Patients were ordered and numbered by increasing planning target volume (PTV) size, and each patient was assigned a unique marker symbol, line pattern, and color for graphical distinction. Table 1 displays these patient characteristics in detail.

**TABLE 1:** Patient characteristics and graphical properties.

Patient number	Fractionation scheme	Liver cancer diagnosis	Location (liver segment)	PTV size (cm^3^)	Graphical symbol
1	40 Gy in 5 Fx	Secondary	III	9.239	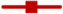
2	40 Gy in 5 Fx	Secondary	VIII	10.303	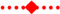
3	40 Gy in 5 Fx	Primary	V/VIII	14.829	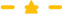
4	48 Gy in 4 Fx	Secondary	III	15.175	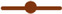
5	40 Gy in 5 Fx	Secondary	I/IV/VIII	24.916	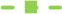
6	48 Gy in 4 Fx	Secondary	I/IV/VIII	33.701	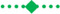
7	48 Gy in 4 Fx	Secondary	I	34.748	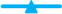
8	40 Gy in 5 Fx	Secondary	II/III	69.648	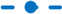
9	48 Gy in 4 Fx	Primary	II/IV	84.925	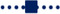
10	40 Gy in 5 Fx	Secondary	V/VIII	100.266	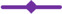

### 2.2 Simulation and reference plan creation

Each patient was simulated on a Philips Ingenia 1.5 T MR simulator (Philips Healthcare, Best, The Netherlands). 4DCT scans were also completed to assess tumor motion. PTV margins varied between 3 and 5 mm when the PTV was an expansion of an internal target volume (ITV). When the PTV was an expansion of a gross tumor volume (GTV) or clinical target volume (CTV), PTV margins varied between 5 and 8 mm. Reference plans were created and optimized in version 6.2 of the Monaco treatment planning system. Beam arrangements varied, but all arrangements avoided treating through high‐Z couch materials and the patient's arms. The sequencing parameters utilized in the reference plans varied among patients based on initial treatment plan template selection and physicist plan optimization efforts.

For the four patients treated with 48 Gy in four fractions, plans were scaled such that 95% of the PTV received the prescription dose (D95% = 48 Gy). For five of the six patients treated with 40 Gy in five fractions, plans were scaled such that 95% of the structure used to define the PTV (i.e., the ITV, CTV, or GTV) received 50 Gy (D95% = 50 Gy), while maintaining PTV coverage of at least the prescription dose (D95% ≥ 40 Gy). For patient 10, the plan was scaled to ensure 100% GTV coverage at 40 Gy (D100% = 40 Gy) while maintaining PTV D95% ≥ 40 Gy. This approach was required due to PTV overlapping with the duodenum. All other dosimetric constraints followed published guidelines.^25^

Hotspots for all patients were evaluated using the near‐maximum dose to 0.03 cm^3^ of the PTV (D0.03cc). For the 48 Gy cohort, hotspots were constrained to D0.03cc ≤ 140% of the prescription dose (≤ 6720 cGy). For patients 1–3 of the 40 Gy cohort, hotspots were constrained to D0.03cc ≤ 150% (≤ 6000 cGy). Patient‐specific hotspot constraints were applied for patients 5, 8, and 10. Due to difficulties achieving ITV coverage, patient 5′s hotspot was constrained to D0.03cc ≤ 157.5% (≤ 6300 cGy), and patient 8′s to D0.03cc ≤ 162.5% (≤ 6720 cGy). For patient 10, the hotspot was limited to D0.03cc ≤ 125% (≤ 5000 cGy) due to PTV‐duodenum overlap. Table 2 lists the features of the 10 reference plans in detail.

**TABLE 2:** Reference plan details.

Patient number	PTV definition	Dose scaling	Hotspot	Max. # of segments per plan	Min. MU/segment	Min. segment width (cm)	Min. segment area (cm^2^)	Fluence smoothing
1	ITV + 5 mm	ITV: D95% = 50 Gy PTV: D95% ≥ 40 Gy	D0.03cc ≤ 150%	70	4.00	0.70	4.000	Medium
2	CTV + 5‐8^a^ mm	CTV: D95% = 50 Gy PTV: D95% ≥ 40 Gy	D0.03cc ≤ 150%	40	4.00	0.70	2.000	Medium
3	GTV + 7 mm	GTV: D95% = 50 Gy PTV: D95% ≥ 40 Gy	D0.03cc ≤ 150%	90	4.00	0.70	2.000	Medium
4	ITV + 0‐5^a^ mm	PTV: D95% = 48 Gy	D0.03cc ≤ 140%	70	4.00	0.70	4.000	Medium
5	ITV + 5 mm	ITV: D95% = 50 Gy PTV: D95% ≥ 40 Gy	D0.03cc ≤ 157.5%	70	4.00	0.70	2.000	Medium
6	ITV + 3 mm	PTV: D95% = 48 Gy	D0.03cc ≤ 140%	50	3.00	0.70	3.000	Medium
7	ITV + 3 mm	PTV: D95% = 48 Gy	D0.03cc ≤ 140%	50	3.00	0.70	3.000	Medium
8	ITV + 5 mm	ITV: D95% = 50 Gy PTV: D95% ≥ 40 Gy	D0.03cc ≤ 162.5%	90	4.00	0.70	2.000	Medium
9	CTV + 5 mm	PTV: D95% = 48 Gy	D0.03cc ≤ 140%	75	3.00	0.70	3.000	Medium
10	ITV + 5 mm	GTV: D100% = 40 Gy PTV: D95% ≥ 40 Gy	D0.03cc ≤ 125%	60	3.00	0.70	4.000	Medium

^a^ Denotes asymmetric expansion.

### 2.3 Experimental plan creation

For each patient, 33 new treatment plans were generated from the reference plans after they had been treated clinically. New plans were created by systematically modifying the following sequencing parameters one at a time using the values listed in Table 3: (1) maximum number of segments per plan, (2) minimum MU/segment, (3) MSW, (4) MSA, and (5) FS. Since the goal of the main work of this study was to evaluate the individual impact of each Monaco sequencing parameter, multiple sequencing parameters were not changed simultaneously. Instead, the four unaltered parameters were kept identical to those in the reference plan. Beam arrangements, dosimetric constraints, and hotspot constraints were unchanged from the reference plans. IMRT optimization criteria were retained from the reference plan except when adjustments were required to meet dosimetric and/or hotspot constraints.

**TABLE 3:** Sequencing parameter values tested.

Parameter	Tested values
Max. # of segments per plan	10, 30, 50, 70, 90, 100
Min. MU/segment	1, 5, 10, 15, 20, 25
Min. segment width (cm)	0.50, 0.75, 1.00, 1.25, 1.50, 1.75, 2.00
Min. segment area (cm^2^)	0.5, 1.0, 2.5, 5.0, 7.5, 10.0, 12.5, 15.0, 17.5, 20.0
Fluence smoothing	Off, low, medium, high

### 2.4 Experimental plan optimization and analysis

Any time a Monaco sequencing parameter is changed, the previously calculated dose distribution and segments are removed from the affected plan, and the plan must be fully re‐optimized. Optimization in Monaco is a two‐stage process.^24^ During stage 1, Monaco generates an optimized fluence distribution based on user‐defined target and organ‐at‐risk constraints. During stage 2, Monaco creates, weights, and optimizes deliverable segments based on the optimized fluence map and sequencing parameters. After the sequencing parameters were altered as previously described, all 330 new plans underwent the full, two‐stage re‐optimization process. The newly calculated doses for all plans were scaled in the same manner as the corresponding reference plans, as described in Section 2.2. After completion of optimization and dose scaling, the following metrics were recorded: MU/Fx, the 100% isodose volume, the 50% isodose volume, optimization stage 1 time, optimization stage 2 time, and estimated delivery time. Optimization times and estimated delivery times were obtained from Monaco log files.

Plan quality was evaluated using the conformality index (CI) and gradient index (RI) as defined in RTOG 0915:

(1) $$CI=\frac{V_{100\%}}{V_{PTV}}\;$$

(2) $$RI=\frac{V_{50\%}}{V_{PTV}}\;$$

where *V*_PTV_ is the PTV volume, *V*_100%_ is the 100% isodose volume, and *V*_50%_ is the 50% isodose volume.^26^ These indices were selected due to their frequent use in the literature and to enable a simple and straightforward comparison of the target coverage (CI) and dose falloff (RI) between the various treatment plans for a single patient.^27^, ^28^ Plans with a CI greater than 1.6 and/or an RI greater than 9.0 were deemed clinically unacceptable based on institutional guidelines. Additionally, any plan with one or more unachievable dosimetric constraints or an unachievable hotspot constraint was deemed clinically unacceptable regardless of CI or RI values.

### 2.5 Combination plan creation

To motivate future work, one additional plan for each patient was created using combinations of altered sequencing parameters based on the results obtained from the main work of this study. All plans used a maximum number of segments per plan of 30, a minimum MU/segment of 25, an MSW of 0.70 cm, and high FS. Plans for patients 1–5 used an MSA of 4 cm^2^ while those for patients 6–10 used an MSA of 15 cm^2^. The individual sequencing parameters selected for these combination plans were chosen to minimize either MU/Fx or optimization stage 2 time without causing unacceptable plan quality, with the expectation that their combination would achieve both outcomes. MSA for patients 1–5 was limited to 4 cm^2^ since this was the largest MSA expected to guarantee a clinically acceptable plan for every patient in this group due to their smaller PTV sizes (less than 30 cm^3^). Like the experimental plans from the main work of this study, all combination plans were generated from the clinically treated reference plans using the same beam arrangements, dosimetric constrains, and hotspot constraints as the reference plans. IMRT optimization criteria were once again retained from the reference plan except when adjustments were required to meet dosimetric and/or hotspot constraints. Following full, two‐stage re‐optimization, the same dose scaling methods used for the experimental plans were also used for the combination plans.

## 3 RESULTS

### 3.1 Maximum number of segments per plan

**FIGURE 1:**
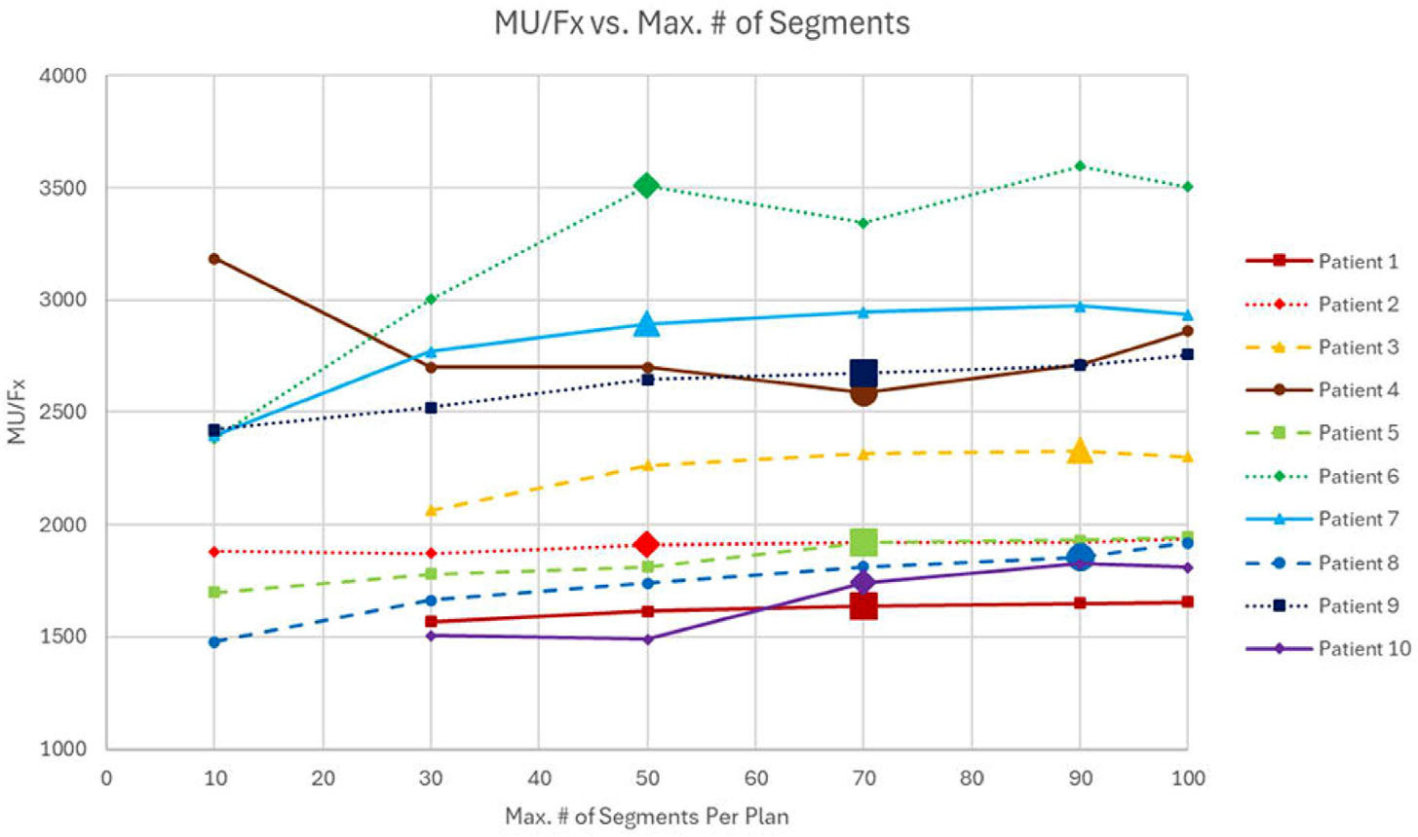
MU/Fx versus maximum number of segments per plan for all patients. The reference values for each patient are delineated by an extra‐large data point.

Figure 1 shows the change in MU/Fx as the maximum number of segments per plan increased for clinically acceptable plans. Excluding an anomalous data point at 10 segments for patient 4, MU/Fx rose by an average of 18.8% (range 3.4%–50.8%) between the minimum and maximum MU/Fx values. As the maximum number of segments increased from 30 to 100, the CI and RI showed small improvements toward lower numbers. CIs improved by 5.7% on average, while RIs improved by 3.4% on average. The exact values for each index depended more on the patient than the maximum number of segments per plan, with CIs ranging from 0.97 to 1.52 and RIs ranging from 4.31 to 8.04. All patients showed worse conformality and dose falloff below 30 segments with CIs increasing by 14.8% on average and RIs increasing by 30.3% on average. For patients 1, 3, and 10, the increase in CI and RI resulted in clinically unacceptable plans. For patient 10, unachievable dosimetric constraints and an unachievable hotspot constraint were also observed. The plan with 10 segments for patient 4 showed the largest increase in RI that did not result in a clinically unacceptable plan (29.3%), so this plan was treated as clinically unacceptable and excluded from the analysis of changes in MU/Fx.

For patients 4 and 6, anomalous decreases in MU/Fx were observed between 50 and 70 segments. Since all plans for patient 4 that altered sequencing parameters other than the maximum number of segments per plan used 70 segments, it was possible to note that the value for MU/Fx observed in Figure 1 for patient 4 at 70 segments was abnormally low when compared to the values observed in other patient 4 plans that used nearly identical values for the other four parameters. Similarly, since all plans for patient 6 that altered the other sequencing parameters used 50 segments, it was possible to note that the value for MU/Fx observed in Figure 1 for patient 6 at 50 segments was abnormally high. Thus, it is likely that the anomalous decreases between 50 and 70 segments observed for these two patients can be attributed to the noise that is characteristic of Monaco's Monte Carlo optimization algorithm.

**FIGURE 2:**
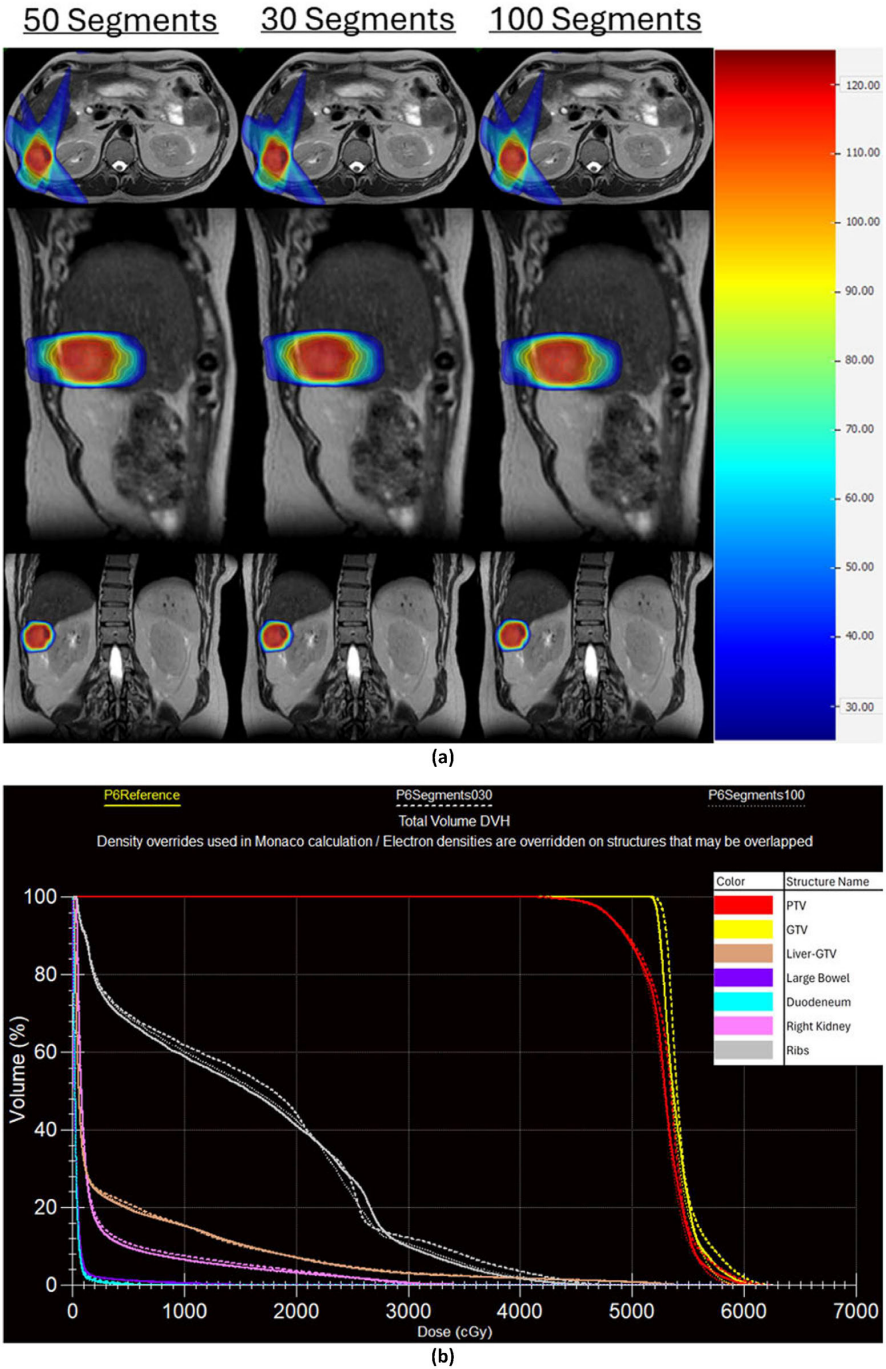
(a) Dose distributions for patient 6 for plans with 50 segments (reference value), 30 segments, and 100 segments. (b) DVH plot for patient 6 for plans with 50 segments (P6Reference), 30 segments (P6Segments030), and 100 segments (P6Segments100).

Changes in the maximum number of segments had no effect on optimization stage 1 time. For patients 6–10 (the five larger PTV patients), optimization stage 2 time rose by an average of 109 ±74 s (148%, range 1.1%–416%) and estimated delivery time rose by an average of 198±48 s (61.5%, range 43.6%–86.7%) as the maximum number of segments increased for clinically acceptable plans. For patients 1–5 (the five smaller PTV patients), optimization stage 2 times showed no clear trends and estimated delivery time rose by an average of 79±54 s (25.5%, range 7.9%–58.6%) as the maximum number of segments increased for clinically acceptable plans. The data for optimization stage 2 times and estimated delivery times is presented in Table 4. Additionally, dose distributions for patient 6 at 30, 50, and 100 segments as well as a DVH comparing those three plans are shown in Figure 2.

**TABLE 4:** Optimization stage 2 times and estimated delivery times at different maximum numbers of segments per plan for each patient. Only clinically acceptable plans are included.

Patient number	Maximum number of segments per plan	Optimization stage 2 time (s)	Estimated delivery time (s)
1	30	129	274
	50	157	306
	70	164	308
	90	160	310
	100	163	308
2	10	49	297
	30	41	317
	50	49	349
	70	57	348
	90	36	351
	100	43	352
3	30	63	352
	50	63	416
	70	41	440
	90	58	461
	100	79	457
4	10	109	507
	30	154	458
	50	203	486
	70	264	496
	90	246	524
	100	266	547
5	10	82	275
	30	113	309
	50	80	341
	70	62	397
	90	58	421
	100	61	435
6	10	51	401
	30	124	519
	50	169	623
	70	197	628
	90	229	696
	100	262	679
7	10	130	383
	30	160	441
	50	131	490
	70	195	520
	90	218	561
	100	238	571
8	10	90	234
	30	148	288
	50	176	333
	70	215	376
	90	224	424
	100	207	437
9	10	97	381
	30	141	400
	50	142	450
	70	173	486
	90	212	525
	100	205	547
10	30	150	262
	50	141	297
	70	172	375
	90	108	416
	100	151	418

### 3.2 Minimum monitor units per segment

**FIGURE 3:**
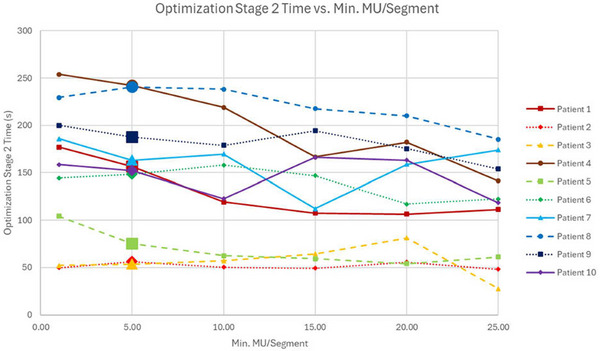
Optimization stage 2 time versus minimum MU/segment for all patients. The reference values for each patient are delineated by an extra‐large data point.

MU/Fx, CI, and RI were negligibly affected by changes in minimum MU/segment. Optimization stage 1 time and estimated delivery time were also negligibly affected. As shown in Figure 3, only optimization stage 2 time varied appreciably with the minimum MU/segment, showing an average decrease of 41±31 s (26.2%, range 3.0%–47.2%) as the minimum MU/segment increased.

### 3.3 MSW

An MSW of 2.00 cm caused plans for patients 1–4 (the four smaller PTV patients) to either be incalculable or to show a factor of two increase in MU/Fx. MU/Fx for patients 5–10 (the six larger PTV patients) was negligibly affected. CI and RI showed similar trends as MSW increased. Patients 5–10 showed only a slight increase in CI and RI at 1.75 cm and 2.00 cm MSW, while patients 1–4 began to show much worse conformality and dose falloff at 1.50 cm MSW. For patients 3 and 4, the increased CI and RI resulted in clinically unacceptable plans at 2.00 cm and 1.75 cm MSW, respectively.

Unachievable dosimetric constraints and unachievable hotspot constraints were observed for patient 3 at 2.00 cm MSW and for patient 4 at 1.50 and 1.75 cm MSW (the plan at 2.00 cm MSW was incalculable for patient 4). Additionally, large values of MSW resulted in the only four plans that were deemed clinically unacceptable based solely on a failed hotspot constraint. MSW of 1.75 cm caused an excessively hot hotspot for patient 2, while MSWs of 1.50, 1.75, and 2.00 cm all caused excessively hot hotspots for patient 8.

Changes in MSW had negligible effects on optimization times and estimated delivery times.

### 3.4 MSA

**FIGURE 4:**
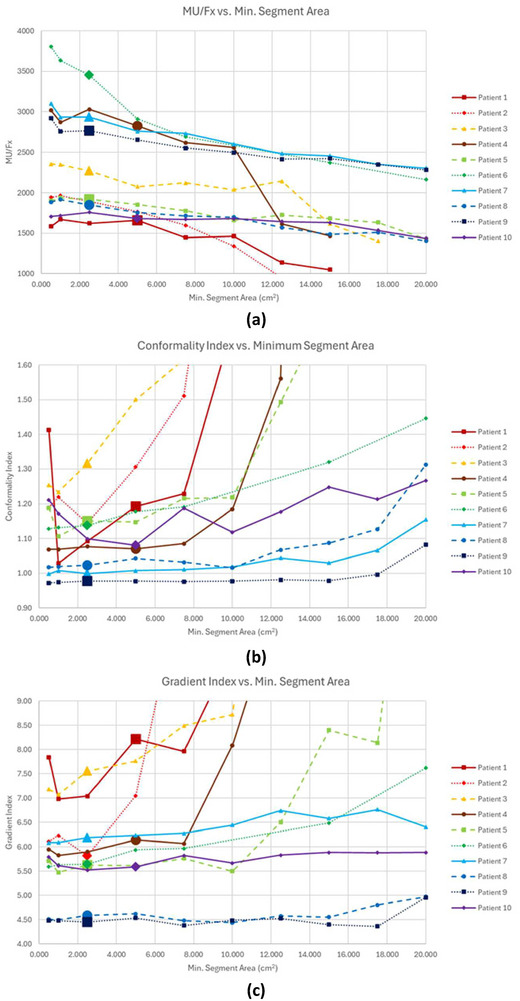
(a) MU/Fx, (b) CI, and (c) RI versus MSA for all patients. Results for patient 2 with fewer than 1000 MU and for patients 1–5 with CI greater than 1.6 or RI greater than 9 are not displayed since these plans were deemed clinically unacceptable. The reference values for each patient are delineated by an extra‐large data point.

As MSA increased, MU/Fx consistently decreased as shown in Figure 4a. Percent decreases between the maximum and minimum MU/Fx values for clinically acceptable plans ranged between 3.7% and 43.2% with an average of 19.1%. Figure 4b shows that CI trends varied as MSA increased. For patients 1–5 (the five smaller PTV patients), CIs rose sharply, and worsening conformality eventually led to clinically unacceptable plans with the exact MSA at which this occurred depending primarily on the patient. For patients 6–10 (the five larger PTV patients), the CI rose more gradually, and conformality remained clinically acceptable for all MSAs. Figure 4c shows that RI trends mirrored CI trends. Plans could not be calculated at 17.5 cm^2^ for patients 1, 2, and 4, or at 20.0 cm^2^ for patients 1–4. Unachievable dosimetric constraints were observed for patient 3 at 12.5 cm^3^ and 17.5 cm^3^, for patient 4 at and above 10.0 cm^3^, and for patient 7 at 20.0 cm^3^.

**FIGURE 5:**
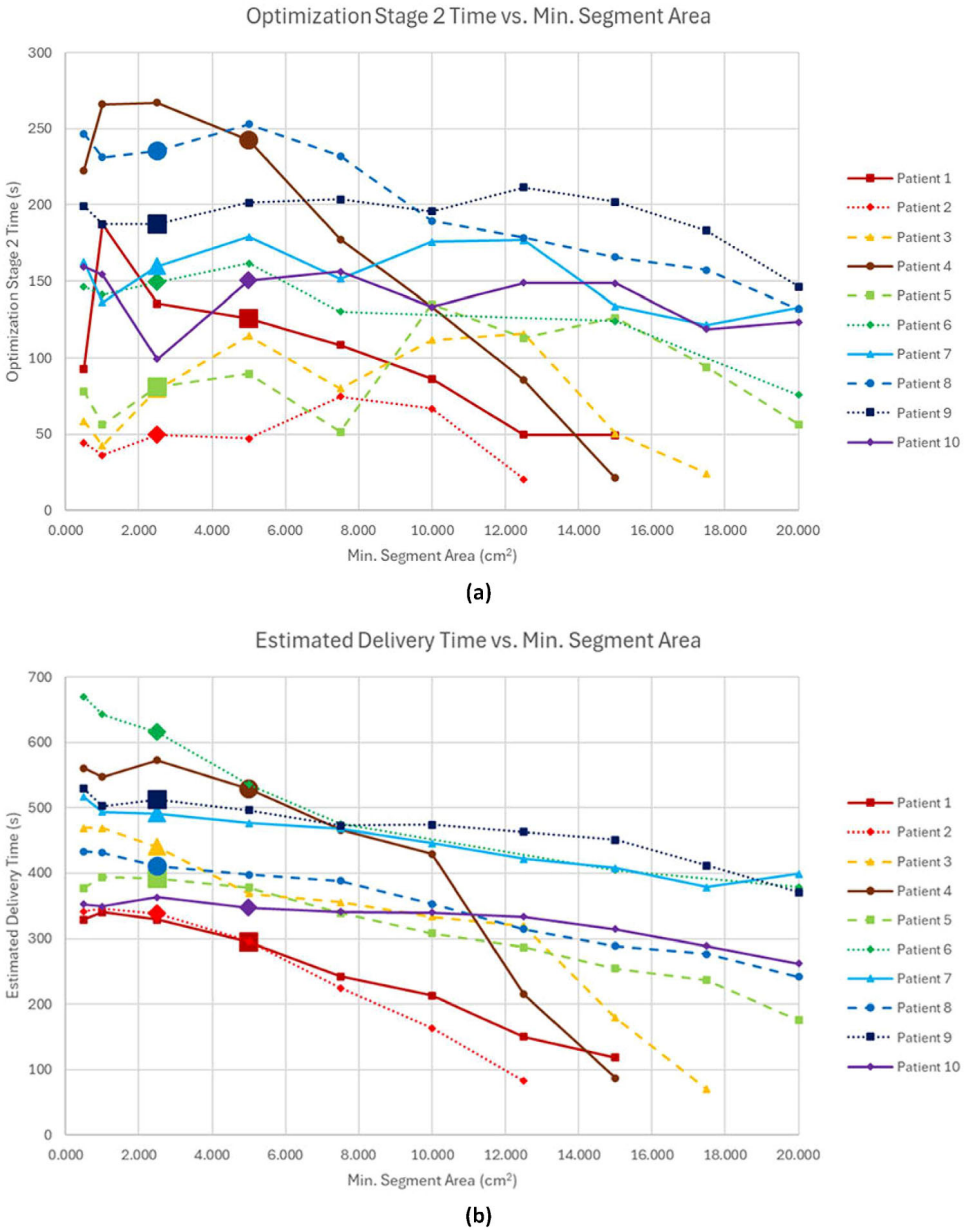
(a) Optimization stage 2 time and (b) estimated delivery time versus MSA for all patients. The reference values for each patient are delineated by an extra‐large data point.

**FIGURE 6:**
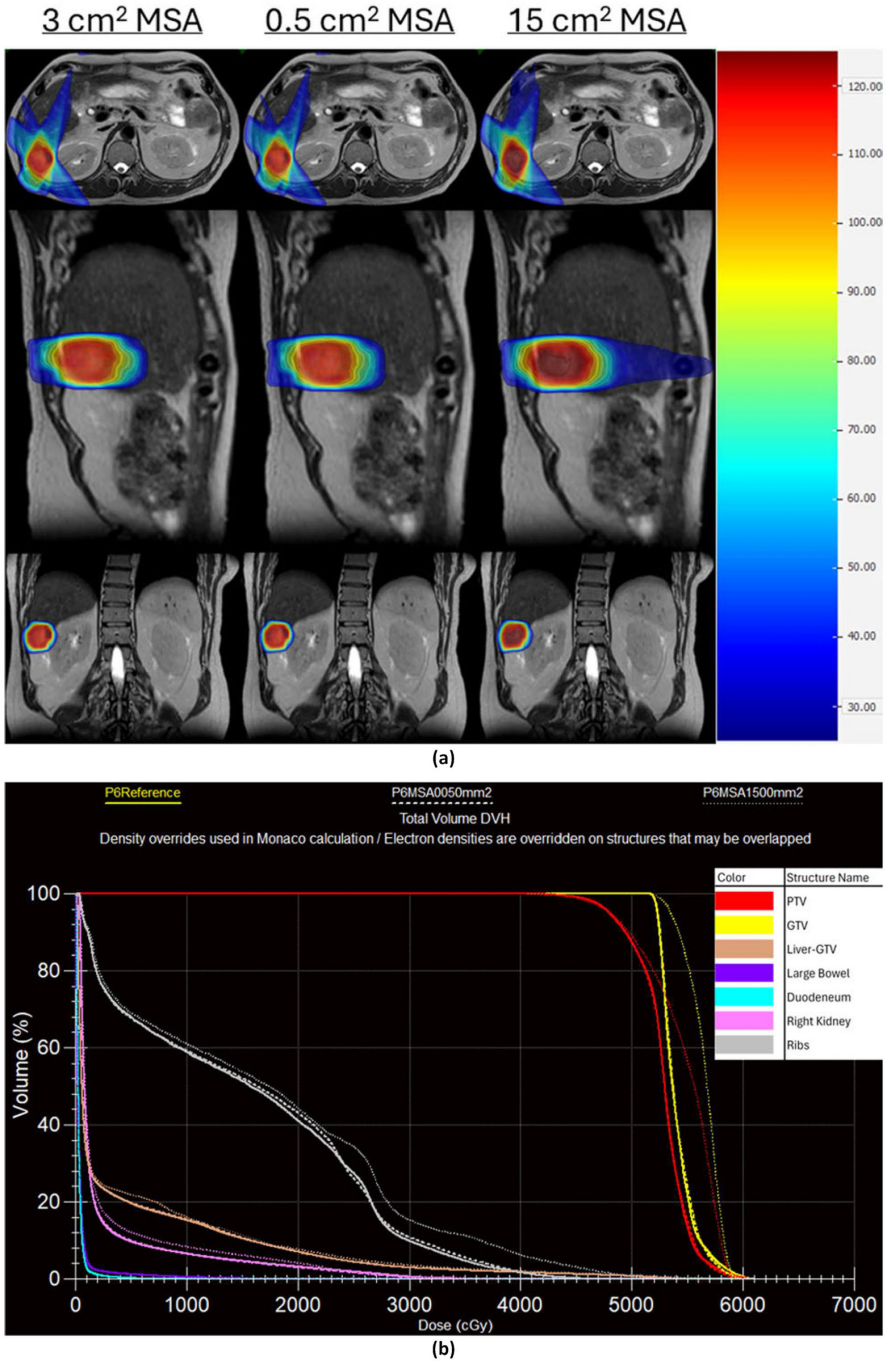
(a) Dose distributions for patient 6 for plans with MSA of 3 cm^2^ (reference value), 0.5 cm^2^, and 15 cm^2^. (b) DVH plot for patient 6 for plans with MSA of 3 cm^2^ (P6Reference), 0.5 cm^2^ (P6MSA0050mm2), and 15 cm^2^ (P6MSA1500mm2).

Changing MSA did not affect the optimization stage 1 time, however, both optimization stage 2 time and estimated delivery time were affected as shown in Figure 5. No clear trend emerged for optimization stage 2 time for the clinically acceptable plans of patients 1–5, however, for patients 6–10, optimization stage 2 time decreased by an average of 63±32 s (33.8%, range 22.5%–48.1%) as MSA increased. For the clinically acceptable plans of all patients, the estimated delivery time decreased by an average of 125±77 s (26.3%, range 6.1%–44.2%) as MSA increased. Dose distributions for patient 6 at 0.5, 3, and 15 cm^2^ MSA as well as a DVH comparing those three plans are shown in Figure 6.

### 3.5 FS

Changing FS from off to low to medium had a negligible effect on MU/Fx. However, from medium to high FS, eight of the 10 patients showed a reduction in MU/Fx. The reduction in MU/Fx ranged between 3.0% and 17.6% with an average reduction of 6.5%. CI and RI were negligibly affected by FS.

**FIGURE 7:**
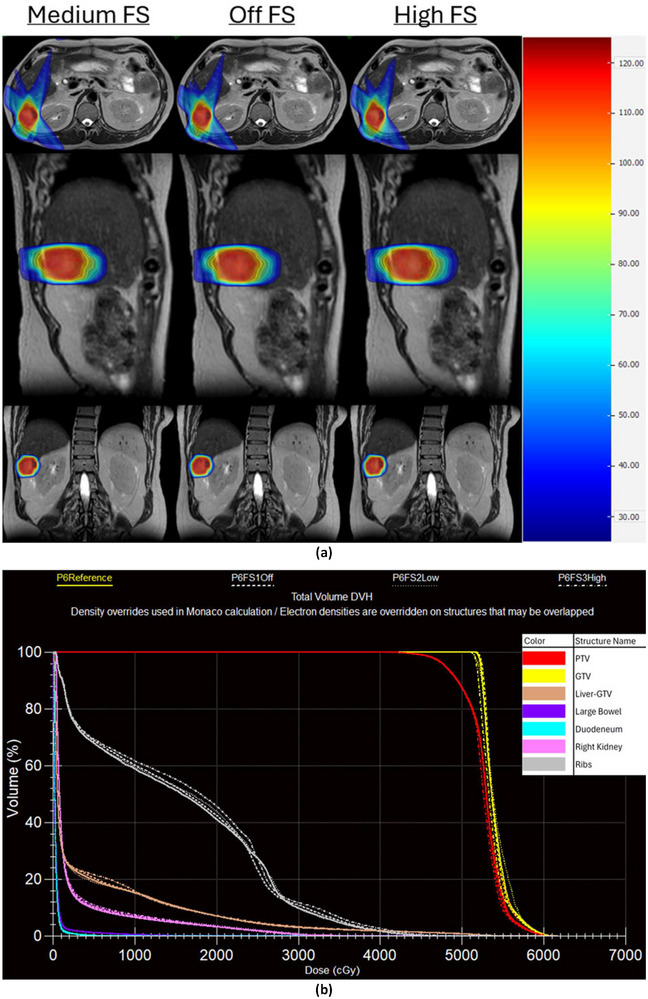
(a) Dose distributions for patient 6 for plans with medium (reference value), off, and high FS. (b) DVH plot for patient 6 for plans with medium (P6Reference), off (P6FS1Off), low (P6FS2Low), and high (P6FS3High) FS.

From medium to high FS, optimization times generally increased, while estimated delivery times generally decreased. Optimization stage 1 times increased by 2.3±3.0 s on average (range −3.0 s–6.5 s) which, although a large percent increase (69.4%), represents a clinically insignificant amount of time. Optimization stage 2 times showed similarly small changes, increasing by 4.3 ±20 s (range −32.0 s–32.6 s) or by an average of 7.9%. The same eight patients who showed a decrease in MU/Fx from medium to high FS also showed a decrease in estimated delivery time. For those eight patients, the reduction in estimated delivery time ranged between 14 and 102 s with an average of 34.3 s (7.4%). Data for MU/Fx, optimization times, and estimated delivery times at medium and high FS is presented in Table 5. Additionally, dose distributions for patient 6 for plans with FS set to off, medium, and high as well as a DVH comparing all four FS settings are shown in Figure 7.

**TABLE 5:** MU/Fx, optimization times, and estimated delivery times at medium and high FS for each patient.

Patient number	Fluence smoothing	MU/Fx	Optimization stage 1 time (s)	Optimization stage 2 time (s)	Estimated delivery time (s)
1	Medium	1641	4	166	308
	High	1503	7	155	279
2	Medium	1939	2	59	340
	High	1817	6	91	326
3	Medium	2318	3	68	459
	High	2204	10	89	415
4	Medium	2654	12	248	511
	High	2799	10	216	517
5	Medium	1931	3	102	397
	High	1844	7	88	371
6	Medium	3445	8	156	616
	High	2839	7	167	514
7	Medium	2880	13	159	490
	High	2795	10	161	471
8	Medium	1857	6	194	420
	High	1774	9	223	394
9	Medium	2711	11	188	501
	High	2627	15	190	486
10	Medium	1723	6	182	362
	High	1857	10	184	369

### 3.6 Relationship between estimated delivery time and monitor units per fraction

**FIGURE 8:**
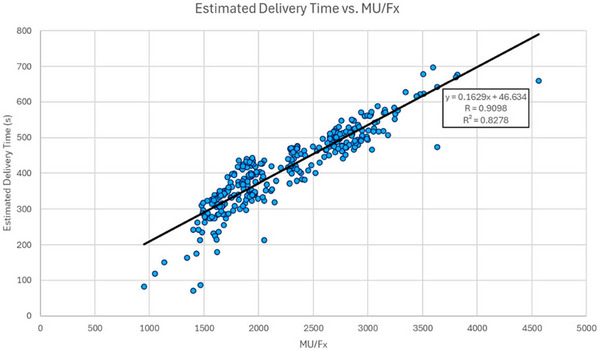
Estimated delivery time versus MU/Fx. Each dot represents an estimated delivery time‐MU/Fx pair from an individual treatment plan. The line represents the best‐fit linear regression.

Figure 8 shows the results of plotting estimated delivery time against MU/Fx for all 315 calculable plans across all 10 patients. Estimated delivery time and MU/Fx are strongly correlated, with linear regression giving an *R* value of 0.9098 and an *R^2^* value of 0.8278. The slope and y‐intercept of the regression indicate that it takes 0.1629 s to deliver 1 MU, and that an average of 46.634 s is spent transitioning between segments during a full treatment delivery.

### 3.7 Combination plans

For all 10 combination plans, all dosimetric and hotspot constraints were satisfied, and all values for CI and RI remained in the clinically acceptable range. MU/Fx values and estimated delivery times were among the lowest achieved throughout the study. Optimization stage 1 times were among the longest seen throughout the study, and the times aligned well with those observed in the main work of this study when FS was set to high. Optimization stage 2 times were below the individual averages observed in the main work of the study for patients 1, 4, and 6–10. For patients 2, 3, and 5, optimization stage 2 times were above the individual averages. The data collected for the 10 combination plans is presented in Table S1.

## 4 DISCUSSION

Although it is not possible to recommend exact values for each sequencing parameter based on the data collected in this study, several consistent trends emerged that can help clinicians make more informed decisions during treatment planning.

The largest reductions in MU/Fx are achieved by reducing the maximum number of segments per plan or by increasing the MSA. For all patients in this study, as long as the maximum number of segments was not lowered below 30, changes in plan quality were minimal and acceptable. This indicates that lowering the maximum number of segments may be a generally useful strategy for reducing the number of MU/Fx. For smaller PTV sizes (less than 30 cm^3^ in this study), increasing the MSA may be less useful since the resulting reduction in conformality and dose falloff can lead to clinically unacceptable plans. A less impactful but still effective alternative is setting FS to high. The reduction in MU/Fx will be less substantial, but there also will not be an appreciable effect on conformality or dose falloff.

It should be expected that changing FS to high would have the smallest effect on MU/Fx. This is because setting smoothing to high does not directly alter segment size or the number of segments Monaco is allowed to use. Instead, it reduces segment complexity by smoothing the ideal fluence distribution that segments are mapped to. This indirectly causes plans that would otherwise be highly modulated to use fewer, larger segments, leading to a smaller reduction in MU/Fx. Similar findings by Jimenez‐Puertas et al. for conventional prostate SBRT further support this conclusion that changing FS to high can have a modest effect on the MU/Fx for highly modulated plans.^20^

Changes to the minimum MU/segment and MSW that resulted in clinically acceptable plans had a negligible overall effect on MU/Fx, CI, and RI. Thus, there is little justification for altering any established institutional defaults for these parameters. Although MSWs of 1.5 cm or greater sometimes increase CI and RI to the point of being clinically unacceptable or cause unachievable dosimetric/hotspot constraints, a reasonably small MSW of 1.00 cm or less should always be a safe choice, and any segment width less than 1.50 cm is likely acceptable. Although other studies have concluded that increasing MSW in Monaco is an effective way to lower MU/Fx for conventional esophageal, gynecological, and left‐sided breast IMRT, this conclusion does not appear to hold for the specific case of liver SBRT on the Unity.^19^, ^20^, ^21^

All optimization stage 1 times measured in this study were under 30 s, making reductions in optimization stage 1 time clinically irrelevant over the course of treatments that may last more than half an hour. In contrast, optimization stage 2 times extended to as long as five minutes which some clinicians may consider worth reducing. Lowering the maximum number of segments per plan or increasing the MSA will lead to noticeable and consistent reductions in optimization stage 2 time for larger PTV sizes (larger than 30 cm^3^ in this study). Increasing the minimum MU/segment would result in smaller reductions in optimization stage 2 time but should prove effective for any PTV size within the range of sizes investigated in this study (approximately 10 cm^3^ to 100 cm^3^). Each of these adjustments reduces the number of segments that Monaco needs to optimize, suggesting that segment count most directly influences optimization stage 2 time. Among these options, lowering the maximum number of segments per plan or increasing the MSA appear particularly valuable because they also reduce MU/Fx. However, because changing the minimum MU/segment has a minimal impact on MU/Fx, CI, and RI, adjusting this parameter can also be considered if treating a smaller PTV or if reducing optimization stage 2 time is a priority.

The strong correlation between estimated delivery time and MU/Fx is expected since delivery time in the absence of gating should depend primarily on the number of MU delivered and the time required to transition between segment shapes.^16^ The *R^2^* value of 0.8278 indicates that approximately 83% of the variability in estimated delivery time can be explained by variation in MU/Fx, with the remaining 17% likely attributable to the time spent transitioning between segments. This analysis verifies the assertion by Fu et al. that the beam‐on time provides the largest contribution to the treatment delivery time for systems with faster leaf speeds and lower dose rates.^16^ It also affirms that lowering MU/Fx can serve as a planning‐level opportunity to lower treatment delivery time.

Further work is needed to determine how altering multiple sequencing parameters at the same time affects MU/Fx, optimization time, and plan quality. Although the combination plans tested in this study showed promising results, it was not possible to determine if the combination of altered sequencing parameters was truly beneficial or if one parameter dominated the effects and made the others irrelevant. Future studies may be able to determine whether specific combinations of altered sequencing parameters produce the most desirable results or if it is best to only alter one parameter at a time as was done in the main work of this study.

It is important to note that the reductions in estimated delivery time observed in this study reflect planning‐level effects under idealized delivery assumptions and do not account for factors such as gating duty cycles, patient motion, and day‐to‐day adaptive workflow inefficiencies. In practice, these factors may substantially increase overall treatment times and may outweigh the MU‐related time differences afforded by altering sequencing parameters. Thus, the findings of this study should be interpreted as identifying a planning‐level opportunity to improve delivery efficiency rather than as a comprehensive solution to prolonged treatment times on the Unity.

## 5 CONCLUSION

This study systematically evaluated the impact of Monaco sequencing parameters on MU/Fx, plan quality, and plan optimization time for liver SBRT delivered on the Elekta Unity MR‐linac. Altering sequencing parameters was shown to offer a practical, planning‐level opportunity to improve treatment delivery efficiency on a patient‐specific basis by reducing MU/Fx and plan optimization time while maintaining acceptable plan quality. Alongside treatment‐level delivery time reduction strategies such as maximizing gating duty cycles, minimizing opportunities for patient motion, and optimizing day‐to‐day adaptive workflows, careful selection of sequencing parameters may serve as a complementary means of minimizing treatment times in support of the efficient clinical implementation of liver SBRT with Elekta's CMM gating technology.

## AUTHOR CONTRIBUTIONS

All authors contributed to the study design and manuscript preparation, review, and editing. Bryan Bates performed plan simulation, data collection, and data analysis. Amanda Schwarz and Guanghua Yan provided supervision, guidance, and feedback for all aspects of the project.

## CONFLICT OF INTEREST STATEMENT

The authors declare no conflicts of interest.

## Supporting information



Supporting Information

## Data Availability

Authors will share data upon request to the corresponding author.
