# Supplementary material for: Impact of Monaco sequencing parameters on monitor units, plan quality, and optimization time for Elekta Unity liver SBRT plans
Source: J Appl Clin Med Phys. 2026 Mar 18;27(3):e70547. doi: 10.1002/acm2.70547 (PMC13093638; doi:10.1002/acm2.70547)
Supplement: Supplementary file 1 — Supporting Information [file ACM2-27-e70547-s001.docx]

**TABLE S1** Combination plan results.

| Patient Number | MU/Fx | Conformality Index | Gradient Index | Optimization Stage 1 Time (s) | Optimization Stage 2 Time (s) | Estimated Delivery Time (s) |
| --- | --- | --- | --- | --- | --- | --- |
| 1 | 1525 | 1.07 | 7.60 | 6 | 95 | 266 |
| 2 | 1699 | 1.11 | 5.48 | 5 | 65 | 284 |
| 3 | 2152 | 1.54 | 7.62 | 10 | 79 | 351 |
| 4 | 2593 | 1.08 | 5.74 | 10 | 145 | 440 |
| 5 | 1861 | 1.21 | 5.79 | 4 | 121 | 322 |
| 6 | 2356 | 1.25 | 7.00 | 7 | 74 | 369 |
| 7 | 2498 | 1.09 | 6.52 | 10 | 105 | 381 |
| 8 | 1538 | 1.21 | 5.31 | 6 | 104 | 252 |
| 9 | 2340 | 1.02 | 4.39 | 11 | 148 | 357 |
| 10 | 1454 | 1.22 | 5.56 | 8 | 131 | 238 |
